# Test-Retest Reliability and Concurrent Validity of a Single Tri-Axial Accelerometer-Based Gait Analysis in Older Adults with Normal Cognition

**DOI:** 10.1371/journal.pone.0158956

**Published:** 2016-07-18

**Authors:** Seonjeong Byun, Ji Won Han, Tae Hui Kim, Ki Woong Kim

**Affiliations:** 1 Department of Psychiatry, Seoul National University College of Medicine, Seoul, Korea; 2 Department of Neuropsychiatry, Seoul National University Bundang Hospital, Seongnam, Korea; 3 Department of Psychiatry, Yonsei University Wonju Severance Christian Hospital, Wonju, Korea; 4 Department of Brain and Cognitive Science, Seoul National University College of Natural Sciences, Seoul, Korea; University of Toronto, CANADA

## Abstract

**Objective:**

We investigated the concurrent validity and test-retest reliability of spatio-temporal gait parameters measured with a single tri-axial accelerometer (TAA), determined the optimal number of steps required for obtaining acceptable levels of reliability, and compared the validity and reliability of the estimated gait parameters across the three reference axes of the TAA.

**Methods:**

A total of 82 cognitively normal elderly participants walked around a 40-m long round walkway twice wearing a TAA at their center of body mass. Gait parameters such as cadence, gait velocity, step time, step length, step time variability, and step time asymmetry were estimated from the low pass-filtered signal of the TAA. The test-retest reliability and concurrent validity with the GAITRite^®^ system were evaluated for the estimated gait parameters.

**Results:**

Gait parameters using signals from the vertical axis showed excellent reliability for all gait parameters; the intraclass correlation coefficient (ICC) was 0.79–0.90. A minimum of 26 steps and 14 steps were needed to achieve excellent reliability in step time variability and step time asymmetry, respectively. A strong level of agreement was seen for the basic gait parameters between the TAA and GAITRite^Ⓡ^ (ICC = 0.91–0.96).

**Conclusions:**

The measurement of gait parameters of elderly individuals with normal cognition using a TAA placed on the body’s center of mass was reliable and showed superiority over the GAITRite^Ⓡ^ with regard to gait variability and asymmetry. The TAA system was a valid tool for measuring basic gait parameters. Considering its wearability and low price, the TAA system may be a promising alternative to the pressure sensor walkway system for measuring gait parameters.

## Introduction

Maintenance of a proper gait is a complex activity involving integration of attention, planning, memory, and other motor, perceptual and cognitive processes. Gait impairment is closely related to higher cognitive control system degeneration. Gait and gait-related motor disturbances occur in all dementia subtypes from its early or preclinical stages [1]. Even in elderly individuals with normal cognition, slow gait speed [2] and worse gait variability [3] predict cognitive decline or dementia. Gait asymmetry is usually found in Parkinson’s and cerebrovascular diseases, which are common causes of dementia. Gait variability and asymmetry are more discriminative measures of gait, as they identify subtle gait disturbances that precede more readily observed spatio-temporal changes such as decreased gait speed [4].

Objective measurements of spatio-temporal gait parameters aid detection of possible gait impairments, study the related cognitive impairment, and quantify effects of therapeutic interventions. The tri-axial accelerometer (TAA) is popular for analyzing gait features in elderly individuals, is lightweight, and is wearable, which enables data collection for a longer duration in unsupervised and real-life conditions. Moreover, it is easy to apply and cost-efficient, making it suitable for detecting and monitoring subtle gait changes.

A TAA system placed on the lower trunk could detect differences in spatio-temporal gait parameters in healthy children [5] and young and older adults [6, 7], and has shown excellent reliability for basic gait parameters in healthy adults and elderly individuals [8–10]. However, the reliability and validity of a TAA system in older adults with normal cognition has been barely studied though it is essential to validate the system before widespread adoption to the future studies on the relationship between gait and cognition. No study to date has measured participant’s cognitive function in a comprehensive manner. Furthermore, the validity and reliability of gait variability and asymmetry have not been well established despite the increasing use of the parameters in researches and clinical studies. They varied considerably with walking distance, analytical algorithms, and data processing techniques. Earlier reports suggest that measurement accuracy improves with longer distance which allow a greater number of steps to be included [11]. However, the required step numbers and the most adequate reference axis to yield reliable measurements have not been determined for gait variability or asymmetry in the TAA system [11, 12].

Therefore, we investigated the test-retest reliability of spatio-temporal parameters measured with a TAA, to determine the optimal number of steps required for obtaining acceptable reliability levels, to compare the reliability with the GAITRite^Ⓡ^ system, to study the level of agreement with parameters derived from GAITRite^Ⓡ^ system, and to compare the reliability and level of agreement between the three reference axes of the TAA, i.e., the anteroposterior (AP), mediolateral (ML), and vertical axes, in cognitively normal elderly individuals.

## Methods

### Subjects

This study enrolled 82 subjects (47 men and 35 women) aged ≥60 years from among the participants of the Korean Longitudinal Study on Cognitive Aging and Dementia (KLOSCAD), which was designed as a population-based prospective cohort study on cognitive aging and dementia in elderly Koreans [13].

All subjects were free from cognitive, psychiatric, and neurologic disorders, serious medical disorders, any history of musculoskeletal system operation, painful conditions, and inadequate vision or hearing that may influence their gait.

### Clinical assessments

Research geropsychiatrists administered the Korean version of the Consortium to Establish a Registry for Alzheimer’s Disease Assessment Battery, which included semi-structured history taking, physical and neurologic examinations, laboratory tests and neuroimaging [14], the Mini International Neuropsychiatric Interview [15], Mini Mental State Examination (MMSE) [16], Unified Parkinson's Disease Rating Scale (UPDRS) [17], Performance-Oriented Mobility Assessment (POMA) [18], Geriatric Depression Scale (GDS) [19], and Cumulative Illness Rating Scale (CIRS) [20]. The diagnosis of normal cognition was made at a consensus diagnostic conference where 3 or more geropsychiatrists participated. At these conferences a full panel of neuropsychological testes and clinical data were reviewed. Global severity of dementia was determined using the Clinical Dementia Rating (CDR). We selected a group of cognitively normal elderly subjects without any evidence of mild cognitive impairment nor dementia with a CDR of 0.

This study was approved by the Institutional Review Board of the Seoul National University Bundang Hospital. All subjects provided written informed consent themselves or via their legal guardians.

### Measurement of gait

Each subject’s gait was measured using FITMETER^Ⓡ^ (FitLifeInc, Suwon, Korea, hereafter FITMETER) and GAITRite^Ⓡ^ (CIR Systems Inc., Havertown, PA, hereafter GAITRite) simultaneously. The FITMETER is a TAA of 3.5 (L) x 3.5 (W) x 1.3 cm (H) weighing 13.7 g. Measurements were made with a sample rate of 32 Hz (range of ±8 G). The data were stored locally on the device and downloaded to a PC via USB. The GAITRite, a portable gait analysis walkway system, measures temporal and spatial gait parameters via an electronic walkway connected to the USB port of a computer. The walkway size was 452 cm (L) x 90 cm (W) x 0.6 cm (H) with an active sensing area of 365 cm (L) x 61 cm (W). The system contains 13,824 sensors placed with a spatial accuracy of 1.27 cm. Measurements were made with a sample rate of 100 Hz.

We fixed a FITMETER to each subject with an elastic band at the level of the 3^rd^-4^th^ lumbar vertebrae, which is the approximate center of mass. Each subject was barefooted and sat in a straight-backed chair which faced a walkway. Then we asked each subject to (1) rise from the chair on observing a start sign, (2) walk straight to the end of a 20-m flat straight walkway, (3) turn around without stopping (4) walk back to the chair at a preferred, comfortable walking speed, and (5) sit in the chair. The time to complete the task was measured with a stopwatch. We placed the GAITRite electronic mat in the middle of the walkway to measure steady state walking. Each subject repeated this procedure three times. The first trial was an exercise to get accustomed to walking protocol and that was not included in further signal analysis. We analyzed the data from the second and third trials. We synchronized the FITMETER and GAITRite clocks by using the PC clock connected to the GAITRite.

### Data processing and analysis

**Fig 1:**
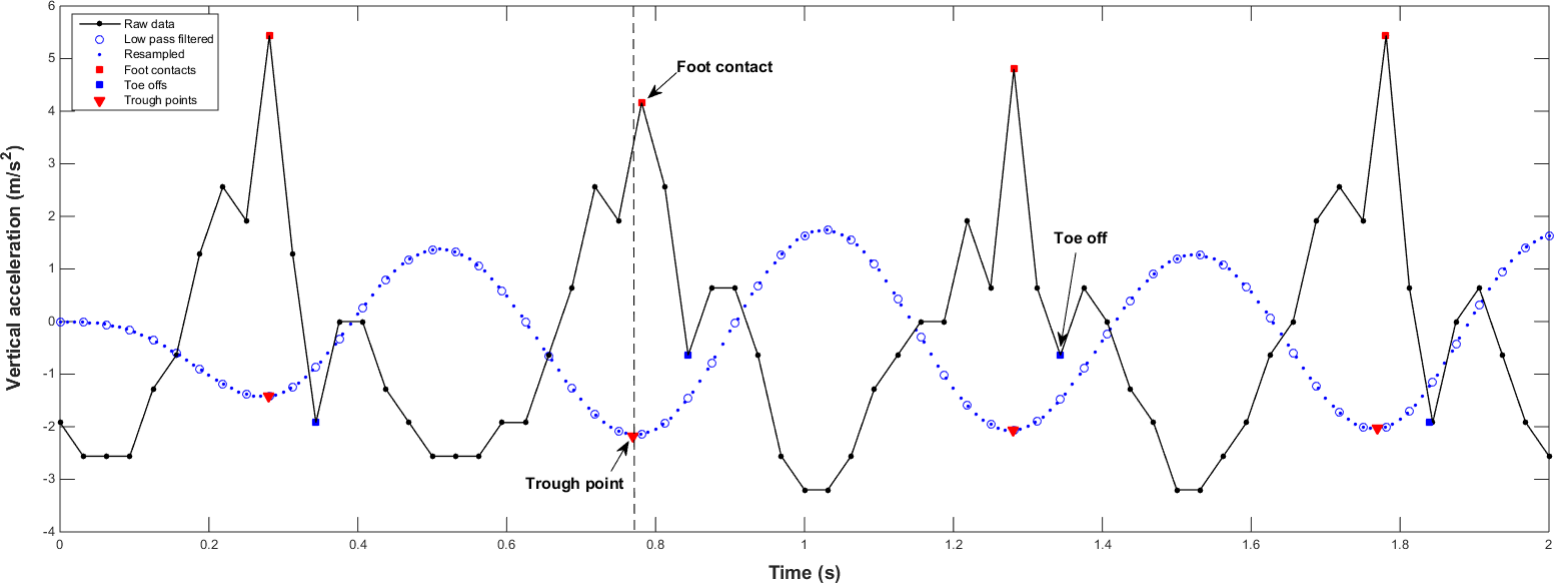
Data processing procedures to detect the instant of foot contact Foot contacts and toe offs are annotated on the raw vertical acceleration data. Trough points are took from processed data (i.e. filtered, interpolated, and resampled data) to estimate the instant of foot contacts.

We read acceleration data as comma separated value (CSV) files using FITMETER manager software (FitLifeInc, Suwon, Korea) and loaded the CSV file into MATLAB (The MathWorks Inc., Natick, MA). We selected signals from the first to the last step for a 20 m straight distance walk. In total, 4 walks of 2 round trials were separately selected for analysis. We applied a low-pass filter (4th order zero-lag Butterworth filter at 2 Hz) to the acceleration data from three axes [21]. Since the acquisition frequency of 32 Hz is a limiting factor in precisely determining the foot contact points, all data was interpolated and resampled at 100 Hz to improve temporal resolution. Several studies have applied the interpolation and resampling to the original acceleration data and shown the data processing steps make it possible to estimate gait parameters validly [22, 23]. After that, we took troughs of the processed data on each axes as the instant of a left or right foot contact for each walk [22]. Procedures to detect foot contacts described above were depicted about vertical acceleration (Fig 1).

We calculated step times using the duration between acceleration troughs. The trough points detected in the central 15 m of a 20-m walking distance were used to measure steady state walking. 2 or more steps from acceleration, and deceleration periods and turning steps were excluded from further analysis manually by visual inspection of the acceleration pattern. As the mean step length of men aged 60–69 was 0.77 m in a prior normative study [24], 20 steps could be sampled from the test distance (15 m/0.77 m≒20). To determine the minimum number of steps required to estimate reliable gait measurements, we sampled from 2 to 20 consecutive steps in the middle of a walk and calculated the respective gait parameters for each data set size.

Cadence was determined from the mean step time (60 s/mean step time). Step time variability quantifies the regularity of gait from one step to the next, and is expressed as the coefficient of variance (CV_%_ = standard deviation/mean x100). To calculate the CV, the scores from the left and right steps were calculated separately and then averaged (CV_Step time_ = [CV_Left step time_+CV_Right step time_]/2). Thus, at least 4 steps (2 from each left and right) were needed to calculate the CV step time. This method avoids confounding step time variability with variation originating from asymmetry between the left and right [12]. Step time asymmetry represents the ratio of the difference between the mean step time of left and right legs to the combined mean step time of both legs. We assigned every even step to one side and odd step to the opposite side without determining each step to be a left or right step. Mean velocity was calculated from the time it took the subject to walk 40 m for each trial. We identified and excluded the sit to first foot contact and last foot contact to sit events at the beginning and at the end of the 40m walk to measure walking time [24]. Mean step length was calculated as the mean step time multiplied by the mean velocity [25]. Inverted pendulum model for estimation of mean step length as described in Zijlstra and Hof [21] was inapplicable to this study because it estimates mean step length only from the vertical acceleration data.

The procedures to calculate the spatiotemporal gait parameters described above were repeated for the ML axis, vertical axis, and the vector summation of the three axes to determine which yields the most reliable and valid measurements.

To obtain more accurate gait parameters from the footprints on the GAITRite mat, we excluded steps placed on the edge of the active sensing area utilizing the associated software. A mean of 4.75 steps were captured on the mat. As subjects passed along the mat 4 times during 2 round trials, a mean of 19 steps (4.75 steps x4) were captured and analyzed. We obtained all gait parameters from the GAITRite software (GAITRite Gold, Version 4.5).

### Statistical analysis

Statistical analysis was performed using SPSS v.19.0 (IBM corp., New York, NY). We compared age, height, weight, leg length, MMSE, CIRS, GDS, UPDRS, and POMA scores between men and women using Student’s t tests. The assumption of a normal distribution was assessed by a Kolmogorov-Smirnov Goodness of Fit test (p<0.05).

To examine the test-retest reliability of the gait parameters obtained from the TAA, we compared the gait parameters obtained in the first and second trials using an intraclass correlation coefficient (ICC) of type (3,1). We combined the data from two walks to construct each trial. To investigate the number of steps required to yield an excellent level of reliability, we repeatedly calculated ICCs while gradually increasing the number of steps analyzed. The reliability was considered as excellent when the ICC was >0.75 and fair-to-good when >0.4 [26].

Absolute agreement of gait parameters obtained from the FITMETER and the GAITRite were determined using ICCs of type (3,1). Relative agreement between the two systems was also established using Pearson’s correlation coefficient. Student’s t-test was used to test the difference between systems’ outcomes (*p* value < 0.05 was considered as significant).

## Results

Subjects’ characteristics are summarized in Table 1. The mean ages were comparable between genders (68.90±6.53 years in men vs. 68.35±5.64 years in women, p = 0.696). Men were taller (p<0.001), heavier (p<0.001), and had longer legs (p = 0.006) than women. Although both men and women were not clinically depressed, women had higher GDS scores (6.40±5.08 in men vs. 10.26±5.24 in women, p = 0.001). The CIRS total score, MMSE, UPDRS, and POMA scores were not different between genders (Table 1).

**Table 1:** Subject characteristics.

Variable	Male (μ±s*d*) [N = 47]	Female (μ±s*d*) [N = 35]	Total (μ±s*d*) [N = 82]	p value
Age	68.90 ± 6.53	68.35 ± 5.64	68.67 ± 6.14	0.696
Height (cm)*	166.03 ± 5.39	154.46 ± 4.94	161.23 ± 7.23	<0.001
Weight (Kg)*	69.57 ± 9.09	59.13 ± 7.24	65.24 ± 9.80	<0.001
Right leg (cm)*	75.65 ± 3.66	73.32 ± 3.46	74.68 ± 3.74	0.005
Left leg (cm)*	75.56 ± 3.72	73.37 ± 3.72	74.65 ± 3.73	0.008
CIRS	4.15 ± 2.28	4.37 ± 2.25	4.24 ± 2.26	0.662
MMSE	27.96 ± 1.33	26.94 ± 2.94	27.52 ± 2.21	0.064
GDS*	6.40 ± 5.08	10.26 ± 5.24	8.05 ± 5.46	0.001
UPDRS	0.04 ± 0.29	0.00 ± 0.00	0.02 ± 0.22	0.391
POMA	27.97 ± 0.17	29.71 ± 0.28	27.94 ± 0.24	0.263

Data are presented as the mean ± standard deviation. CIRS, Cumulative Illness Rating Scale; MMSE, Korean version of Mini Mental Status Examination; GDS, Korean version of Geriatric Depression Scale; UPDRS, Unified Parkinson's Disease Rating Scale; POMA, Performance-Oriented Mobility Assessment

* p<0.05

### Test-retest reliability

**Fig 2:**
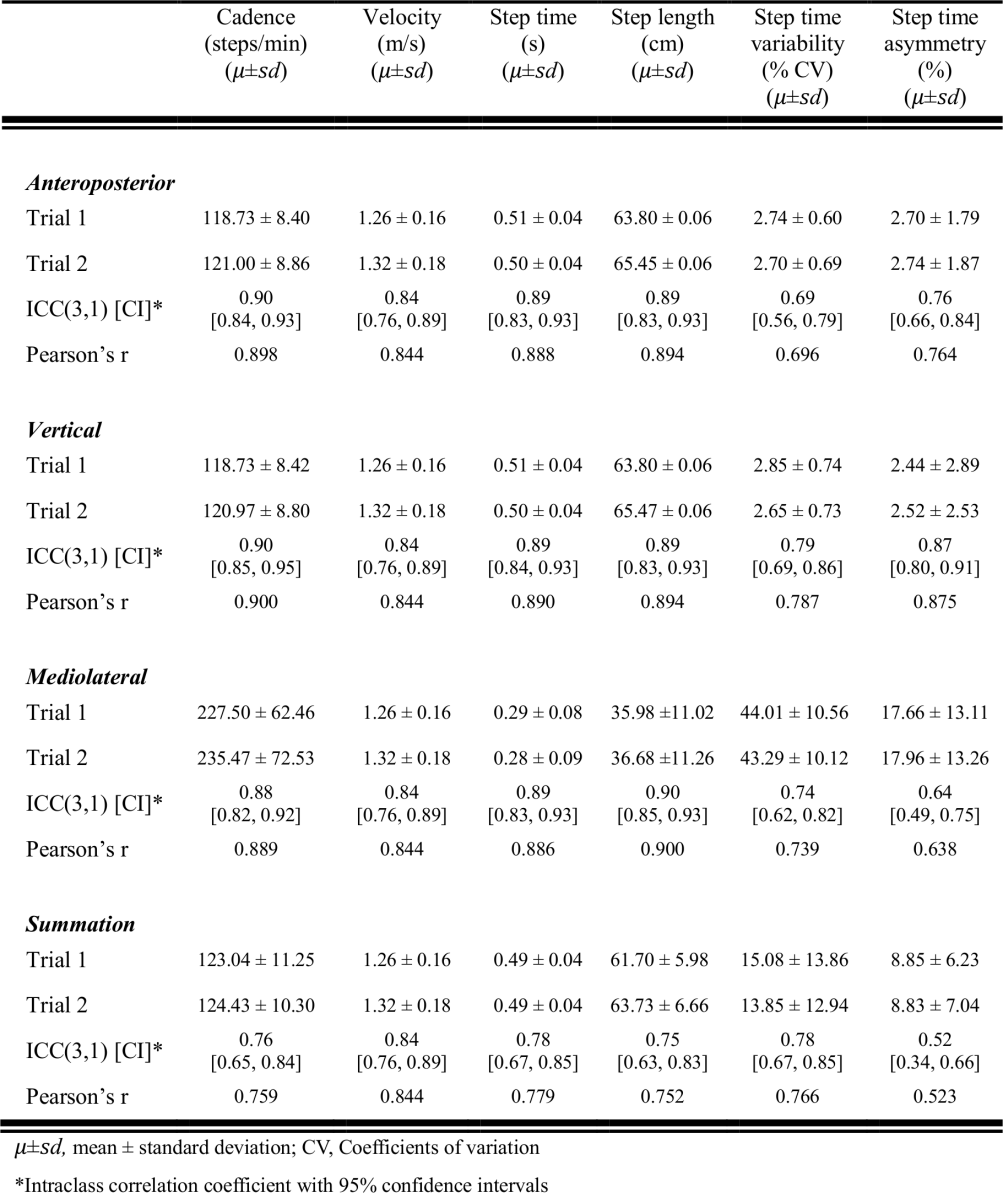
Test-retest reliability of the triaxial accelerometer system for spatiotemporal gait parameters.

Basic gait parameters such as cadence, velocity, step time, and step length showed excellent test-retest reliability in all three axes (ICC = 0.84–0.90) (Fig 2). Data from the vector summation yielded lower but still good reliability (ICC = 0.75–0.84). The reliability of CV of step time which represents gait variability and step time asymmetry varied across the axes. The vertical axis displayed the most reliable results for the CV of step time and step time asymmetry. Overall, gait parameters based on the vertical axis could be measured most reliably, and the reliability of all gait parameters was excellent (Fig 2).

Basic gait parameters measured using GAITRite also showed excellent test-retest reliability (ICC = 0.95 [0.92, 0.97] for cadence, ICC = 0.93 [0.89, 0.95] for velocity, ICC = 0.95 [0.92, 0.97] for step time, ICC = 0.94 [0.91, 0.96] for step length). However, the CV of step time (ICC = 0.09 [-0.13, 0.30]) and step time asymmetry (ICC = 0.06 [-0.16, 0.27]) from the GAITRite were much less reliable than those from the TAA.

**Fig 3:**
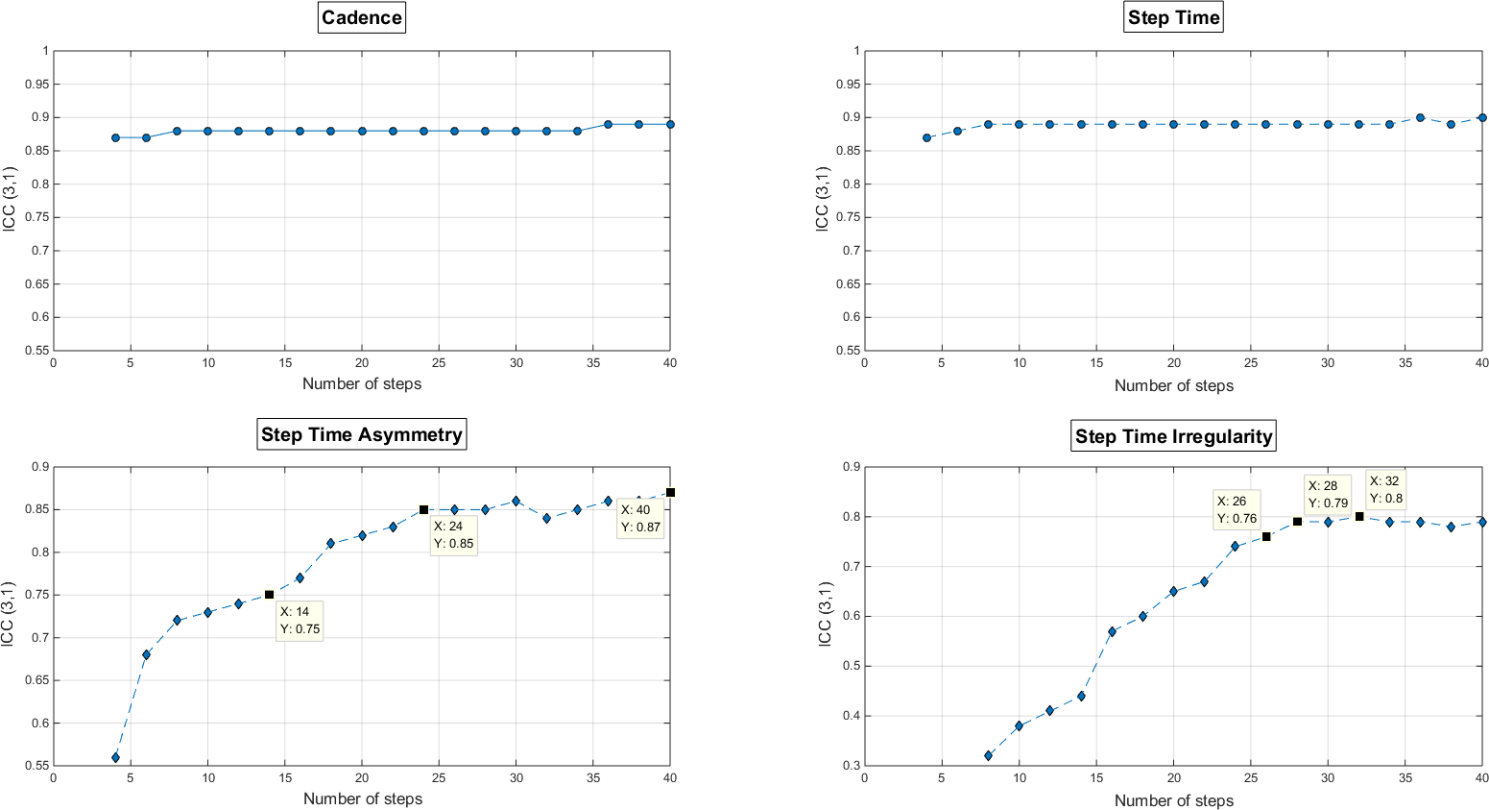
Test-retest reliability of spatiotemporal gait parameters according to the number of analyzed steps Reliability is measured by the intraclass correlation coefficient type of (3,1), or ICC(3,1). The 3 cursor boxes in a graph represent the points to reach ICC = 0.75, plateau of ICC scores, and maximum ICC score, in that order from left to right.

To measure gait parameters using a TAA with excellent reliability (ICC≥0.75), we needed only 4 steps for the basic gait parameters. However, for the CV of step time, we needed 26 steps to achieve an ICC of 0.75 or higher (ICC = 0.76 [0.65, 0.84]), and it reached a plateau at 28 steps (ICC = 0.79 [0.69, 0.86]). For step time asymmetry, we needed 14 steps to achieve an ICC of 0.75 or higher (ICC = 0.75 [0.63, 0.83]), and it reached a plateau at 24 steps (ICC = 0.85 [0.78, 0.90]) (Fig 3).

### Level of agreement between TAA and GAITRite system

**Fig 4:**
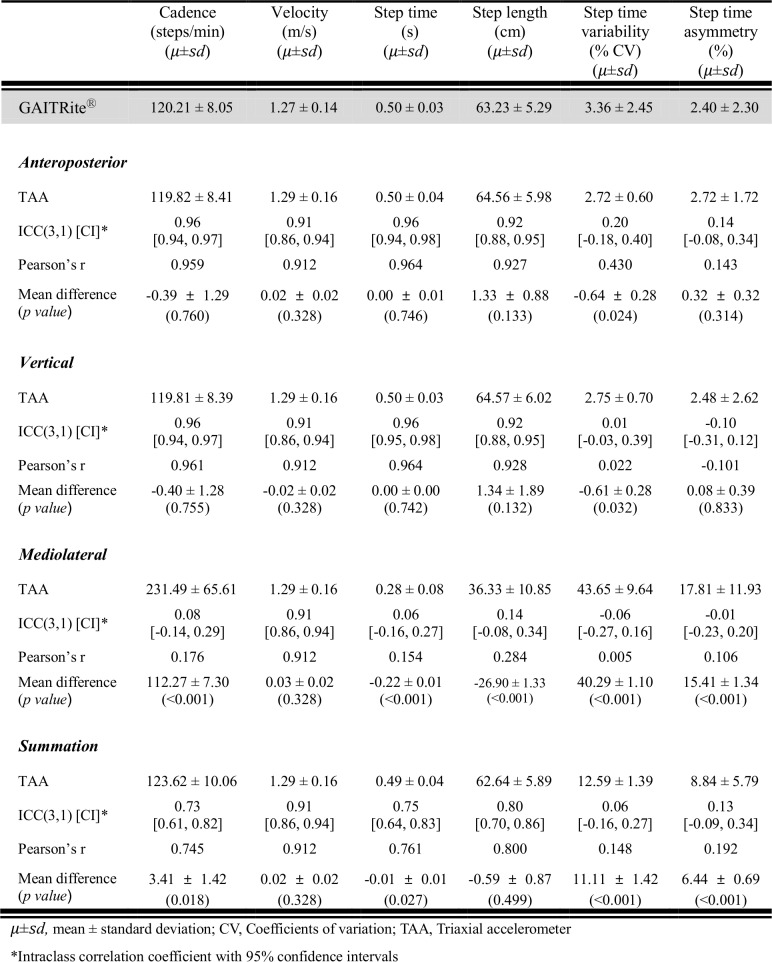
Level of agreement between a triaxial accelerometer and GAITRite^Ⓡ^ for spatiotemporal gait parameters.

Gait parameters estimated by the acceleration data from the vertical or AP axes showed a better level of agreement with the GAITRite than those from the ML axis or vector summation. When the acceleration data from the vertical axis was utilized for gait analysis, the level of agreement with the GAITRite was excellent for the basic gait parameters (ICC = 0.91–0.96), but poor for CV step time (ICC = 0.01 [-0.03, 0.39]) and step time asymmetry (ICC = -0.10 [-0.31, 0.12]). In the case of the AP axis, the results were similar to those from the vertical axis. However, the acceleration data from the ML axis resulted in a poor level of agreement with the GAITRite, even for the basic parameters (Fig 4).

## Discussion

This study demonstrated that excellent test-retest reliability of spatio-temporal gait characteristics measured using a TAA in cognitively and physically normal elderly individuals. Reliability was best with the acceleration data from the vertical axis and validity was best with the acceleration data from the AP axis. The reliability of step time variability and asymmetry improved as the number of steps included in the analysis increased, and it reached an excellent level when 26 or more steps were included for step time variability and 14 steps or more for step time asymmetry. The level of agreement between the TAA and GAITRite was excellent for basic gait parameters while it was poor for step time variability and asymmetry.

The vertical acceleration data of the TAA displayed excellent test-retest reliability for estimating cadence, step time, step length, step time variability, and step time asymmetry. Test-retest reliability of cadence, step time, and step length estimated by the TAA system has been consistently reported to be good [8–10]. However, only a few studies have investigated the reliability of gait variability measured by the TAA system. Hartmann et al. reported excellent reliability for step time variability (ICC = 0.78) in healthy elderly adults, consistent with our observation. In contrast, the reliability of step time variability estimated using the GAITRite was very low (ICC = 0.09) in this study. The reliability of gait variability estimated using the GAITRite is reported to vary depending on the length of the active area on the walkway used for measuring the gait. The step time reliability was good when longer walkways were used. The European GAITRite network group recommends the highest number of gait cycles possible from a practical standpoint, with a minimum of 6 consecutive gait cycles (i.e., a total of 12 consecutive steps) to evaluate stride time variability [27]. The length of the active area on the walkway was too short to catch enough consecutive steps in the present study (3.65 m) and in a previous study that reported low reliability (≤3.5 m) [28]. Similar to the GAITRite, reliability of the TAA based gait analysis improved as more steps were included in the analysis. To achieve an excellent reliability for step time variability and step time asymmetry using a TAA, 26 steps and 14 steps were needed, respectively. However, these numbers are fewer than those in the pressure-sensor walkway system (30–370 steps) [11, 12]. Therefore, our results indicate that gait variability estimated by the TAA system may be comparable to or even better than that by the GAITRite system short walkway. The TAA system may be a promising alternative to the pressure-sensing walkway system for measuring gait due to its reliability in addition to its wearability.

Further, FITMETER measured step time asymmetry more reliably than the GAITRite (ICC 0.87 vs. 0.06) in this study. In contrast, the reliability of step time asymmetry measured using the GAITRite was excellent (ICC>0.9) in a previous study [29]. This discrepancy might be attributed to differences in the participant characteristics. Inter-individual variance in gait asymmetry in the current study must be much lower than that in the previous study, because the participants in the present study were physically and cognitively healthy elderly individuals, whereas the previous study employed chronic stroke patients with a diverse range of gait asymmetry. Since the ICC score is more likely to increase as the between-subject variance increases (ICC = S^2^_between_/[S^2^_between_+S^2^_within_]), we can assume that the FITMETER may reliably measure the gait of stroke patients like those of normal elderly individuals. However, the GAITRite could not measure step time asymmetry as reliably as the FITMETER. Din et al. [30] also found a body-worn monitor like a TAA to be more likely sensitive for measuring gait asymmetry and variability than a pressure-sensor walkway because it measures gait as a continuous activity rather than discrete footfall events.

Agreement levels between the estimated basic gait parameters obtained with the FITMETER and GAITRite were excellent (ICC = 0.91–0.96) when AP or vertical acceleration data were used for the estimation, which is consistent with previous studies (ICC = 0.78–1.00). This result demonstrated excellent concurrent validity of the TAA with regards to basic parameters. Interestingly, the ML acceleration data displayed poor levels of agreement even in basic parameters. The signals from AP, ML and vertical axis were low-pass filtered with a cut-off of 2 Hz, as we expect few movements to contain information of interest above 2 Hz. And the trough points from the filtered acceleration data were used to calculate step times. The poor level of agreement suggests the step detection technique presented above is not applicable to the ML axis.

In contrast to basic parameters, agreement levels between the estimated gait variability and asymmetry between FITMETER and GAITRite were poor (ICC = -0.06–0.20 for step time variability; ICC = -0.01–0.14 for step time asymmetry), which concurs with previous studies (ICC = 0.35–0.51) [30, 31]. Possible explanations for the poor agreement between the two systems have been discussed including misalignment due to device orientation/placement, poor synchronization, difference in sampling rates, drift due to integration, and false allocation of left/right steps. The misalignment and synchronization problems also existed in this work. However, the most probable explanation for the differences may be that the GAITRite used in the present study could not estimate those parameters well. Since the parameters were not measured reliably by the GAITRite, the data could not be valid. Galna et al. [12] suggested that at least 30 steps are required to estimate gait variability, although 50 or more steps are preferable. However, only 19 steps from the GAITRite were included for analysis due to the short length of the walkway in the present study. In addition, Del din at el. [30] reported that the agreement between the TAA and GAITRite systems was good for patients with clear gait asymmetry but poor for normal subjects without gait abnormalities. Collecting acceleration data from a straight walkway system which is long enough to capture 12 consecutive steps or more has practical limitation in space for usual clinical settings. Moreover, gait analysis of normal subjects without gait abnormality will have been conducted in many studies which investigate the different gait features between the normal and study groups. Considering these, we suggests that a TAA may be a better tool for measuring gait variability or asymmetry than a GAITRite especially when limitation in space exists or homogeneous study population with regards to gait such as normal elderly subjects is evaluated. However, further researches still remain to be done on the concurrent validity of gait variability and asymmetry derived from a TAA in normal population using an ideal golden standard tool.

This study has some limitations. First, the sampling rate of the FITMETER was low, and thus temporal aliasing may exist [32]. However, devices with 32-Hz sampling rate have been adopted in several studies because 95% of the power of normal gait is contained below 15 Hz, which requires the sampling rate of 30 Hz or more to measure gait properly [33]. Therefore, the sampling rate of 32 Hz would result in temporal aliasing, but the extent of error would be acceptable. Furthermore, lower sampling rate requires less battery consumption and data storage, which makes it possible to monitor gait for longer duration in real life conditions. In our study, sampling at 32-Hz-frequency and data processing afterward calculated gait parameters with excellent reliability for all parameters and excellent validity for basic parameters, which would be meaningful results from a practical standpoint. Second, inclination of the TAA was not considered. The TAA attached over the trunk could be tilted due to posture and motion. Third, the GAITRite system was not a gold standard for validating the TAA system with respect to gait variability and asymmetry. Therefore, the level of agreement could be interpreted as the concurrent validity only for the basic gait parameters.

In conclusion, the measurement of gait parameters of normal elderly individuals using a single TAA was reliable. The TAA system was a valid tool for measuring basic gait parameters. It has some advantages over the walkway system for measuring gait variability and asymmetry. The TAA system can be a promising alternative to pressure sensor walkway system and perhaps, the 3D motion analysis system because reliable and valid gait measurements can be obtained for longer durations in unsupervised and real-life conditions.
